# Farm animal models for the study of the reproductive features of polyendocrine metabolic ovarian syndrome

**DOI:** 10.3389/fendo.2026.1863933

**Published:** 2026-09-15

**Authors:** Rodrigo A. Carrasco, Dinesh Dadarwal

**Affiliations:** 1 Department of Veterinary Biomedical Sciences, Western College of Veterinary Medicine, University of Saskatchewan, Saskatoon, SK, Canada; 2 Department of Large Animal Clinical Sciences, Western College of Veterinary Medicine, University of Saskatchewan, Saskatoon, SK, Canada

**Keywords:** animal models, farm animal models, hyperandrogenemia, luteinizing hormone, PMOs, testosterone, ultrasonography

## Abstract

Polyendocrine metabolic ovarian syndrome (PMOS) is a multifactorial disease representing one of the most prevalent causes of infertility in women, affecting up to 15% of females of reproductive age. PMOS is characterized by a combination of ovarian dysfunction, hyperandrogenemia and endocrine derangement, frequently accompanied by metabolic comorbidities including insulin resistance and obesity. Despite its clinical importance, the mechanisms underlying its pathogenesis remain incompletely understood. Rodent models have been foundational for genetic and mechanistic studies but differ from humans in fundamental aspects of reproductive physiology and neuroendocrine rhythms may not recapitulate the PMOS phenotype at either the ovarian or the neuroendocrine level. This review evaluates current farm animal models of PMOS in humans with a special emphasis on ovarian and endocrine function. Farm animal species, particularly sheep, cattle, horses and pigs, offer complementary advantages compared to rodents: comparable estrous cycle length, well-characterized follicular wave dynamics, and the capacity for longitudinal endocrine and ultrasonographic monitoring at a scale not feasible in human studies. While no current animal model parallels PMOS in its entirety, domestic animals may be useful to disentangle the mechanisms that are participating in PMOS at the ovarian and endocrine level, such as follicular arrest and hormonal dysregulation. The adoption of farm animals in the study of PMOS will complement mechanistic and clinical studies for future diagnosis and therapy.

## 1 Introduction

Infertility is defined as the inability to conceive after a period of 12 months and affects around 17% of women globally, representing a substantial public health burden (1). Polyendocrine metabolic ovarian syndrome (PMOS, formerly known as polycystic ovarian syndrome) is the most prevalent cause of infertility in women of reproductive age, estimated to affect 15% of this population (2). PMOS is a complex, multifactorial syndrome characterized by ovarian dysfunction, hyperandrogenism and endocrine imbalance, frequently accompanied by metabolic comorbidities (3). Despite its prevalence and the volume of research dedicated to it, the pathogenesis of PMOS remains incompletely understood, and current treatments remain largely symptomatic.

Animal models are indispensable for advancing mechanistic understanding of PMOS beyond what is achievable in clinical studies alone. Recently developed rodent models promise important future contributions: the letrozole mouse model reproduces both reproductive and metabolic features of PMOS (4), and the spontaneously diabetic Goto-Kakizaki rat displays all Rotterdam diagnostic criteria (5). However, rodents differ fundamentally from humans in reproductive physiology. They are polyovulatory, cycle every four to five days, and lack a true luteal phase outside of pseudopregnancy (6). The preovulatory luteinizing hormone (LH) surge in rodents is entrained to a nocturnal circadian rhythm and occurs invariably at the onset of darkness on the day of proestrus (7), a pattern that does not apply to women or most domestic species (8, 9). A recent study in transgenic mice have further questioned whether current rodent models fully recapitulate the neuroendocrine features of PMOS in human (10), highlighting the need for complementary experimental systems.

Farm animals offer several specific advantages for PMOS research. Their estrous cycles are substantially longer and more comparable to the human menstrual cycle, approximately 21 days in cattle, pigs and mares, 17 days in sheep, than those of rodents. Monovulatory farm animal species, including cattle (11), sheep (12) and horses (13), share with women a well-characterized pattern of follicular wave dynamics (14), comparable gonadotrophin secretion profiles, and the amenability to longitudinal monitoring using transrectal (in farm animals) or transvaginal (in women) ultrasonography and repeated blood sampling without compromising normal physiology (15). Ovarian follicular development has been described to follow a wave-like pattern that has been identified in mare, cows, sheep, goat, camelids, wapiti, monkeys and women. The mechanistic basis of follicular wave dynamics, dominant follicle selection, and neuroendocrine regulation of the hypothalamic–pituitary–ovarian axis across domestic species and women is reviewed comprehensively in a companion article (16). The present review focuses specifically on how farm animal species have been and may be used to model PMOS.

The objective of this review is to evaluate the strengths and limitations of farm animal models of PMOS with emphasis on three core PMOS features: ovarian dysfunction, hyperandrogenemia and gonadotropin derangement. We will examine both induced and naturally occurring models, assess which PMOS features are recapitulated consistently, and identify research gaps that large-animal approaches are best positioned to address.

## 2 Follicular dynamics in farm animals and women: unifying concepts and species differences

The traditional division of the menstrual cycle into follicular and luteal phases was established based on which steroid hormone (i.e., estrogen vs progesterone) predominated in circulation during this period. The underlying follicular development throughout the menstrual cycle remained poorly characterized given the lack of available research tools to evaluate ovarian follicular development. The first theory synthetizing the patterns and mechanism of follicular growth (the “propitious moment” or “continuous growth” theory) postulated that follicles display continuous and random growth during the cycle and the largest follicle ovulated because it was mature at the time of the gonadotrophin surge (15). A report using histological evaluation of bovine ovaries during the estrous cycle in the 1960s challenged the propitious moment theory by documenting that “follicles ≥ 5 mm show growth waves during the estrous cycle of cows” (17). The introduction of the non-invasive assessment of ovarian function via ultrasonography was a key development for testing the wave-pattern hypothesis. The ultrasonic evaluation of ovarian follicles takes advantage of the interphase between the echogenicity of follicular wall and the anechogenicity of follicular fluid, which provides an excellent contrast for the detection and accurate quantification of ovarian follicle number and diameter (18). Additionally, ultrasonography enables repeated non-invasive examination of ovarian function, which coupled with serial blood sampling provides a powerful approach to characterize ovarian and reproductive cycles. The wave pattern theory was formulated after a series of pioneer studies using transrectal ultrasonography as a research tool in farm animals in the 80’s and 90s decade and remains the basis of the current reproductive knowledge today (11, 19–21).

Folliculogenesis comprises the development of ovarian follicles from follicle assembly to ovulation, which in the bovine species the progression from primordial follicle to ovulation has been estimated to take 60–90 days (22–24). While a large part of folliculogenesis occurs in a relative gonadotrophin-independent manner, in the final phase (8–10 days) antral follicles acquire follicle stimulating hormone (FSH)- and LH-dependency, followed by a period of exponential growth (25). The follicular antrum develops in secondary follicles reaching 0.3-0.4 mm of diameter and, from this point, the pattern of follicular growth remains poorly characterized until the follicle reaches 2–3 mm of diameter. In bovine ovaries, the growth phase from ≥3 mm of diameter is characterized as a wave-like pattern, where a cohort of follicles grow at a similar rate until one (in monotocous species) or several (in polytocous species) follicles maintain their growth rate (i.e., dominance) while the rest of the cohort diminishes in size and undergo atresia (subordinate follicles). In this context, the detection of a group of follicles ≥3mm is denominated wave emergence and the process of establishing dominance is denominated selection. The emergence of each follicular wave is preceded by enhanced FSH secretion from the pituitary (20, 26) and the selection of a dominant follicle in cattle and mares is based on the selective suppression of FSH by the largest follicle of the wave, restricting the FSH support for the rest of the cohort (26). At the time of selection, the largest follicle exerts a negative feedback loop over hypothalamic and pituitary output by the secretion of follicular products such as estradiol and inhibin, reducing FSH circulating concentrations (27, 28). The reduced levels of circulating FSH, critically supports the growth of the dominant follicle, but not for subordinate follicles. Concomitantly, circulating LH increases transiently just before the moment of deviation, and along with the increases of LH receptor expression in the dominant follicle wall at the moment of deviation, LH pulsatility is responsible for dominant follicle growth (26). After follicular dominance is established, the dominant follicle continues to grow until its growth rate stagnates (static phase) at a follicular diameter that is compatible with ovulation. The fate of the dominant follicle will depend on the occurrence of the preovulatory LH surge which is promoted by increases in circulating estradiol (form the same dominant follicle) in the absence of the inhibitory effect of progesterone.

While differences in follicular sizes and numbers are species-specific, the events during the follicular wave remain remarkably similar in farm animals and women. For instance, in women, the sudden ultrasonographic detection of multiple follicles ≥5 mm is documented as the emergence of a follicular wave, while in cows wave emergence is usually defined as the detection of multiple follicles 3–4 mm diameter. In mares and sheep, wave emergence is usually the detection of several follicles measuring 6 and 2–3 mm, respectively. Selection occurs when the largest follicle reaches 10 mm, 8 mm and 25 mm in women, cow and mare, respectively. The dominant follicle acquires the capacity for ovulation when it reaches the static phase at 12–14 mm, 6–7 mm, 18–20 and 28 mm for cow, sheep, women and mares, respectively (12, 15, 26, 29, 30). The number of follicular waves per estrous or menstrual cycles determines the length of the cycle and are also species-specific. For instance, cattle and sheep show 2–3 and 3–4 follicular waves during the inter-ovulatory interval, respectively. A striking phenomenon in follicular dynamics in women and mares is the existence of major and minor follicular waves throughout the menstrual and estrous cycle. In both cases, a follicular wave emerges and the follicles grow until deviation but only in major follicular waves a dominant follicle is selected (29). Interestingly, in both mares and women the ovulatory follicle emerges within a major wave during the mid estrous or menstrual cycle. In summary, ovarian function in farm species provide a reliable and useful approach to understand biological mechanisms of reproductive biology in women (**Table 1**).

**Table 1:** Comparative summary of reproductive characteristics and ovarian events in domestic animals and human.

Reproductive characteristics	Cow	Sheep	Mare	Woman
Follicular diameter at wave emergence (mm)	3 – 4 (20)	2-3 (12, 31)	6 (30, 32)	4-5 (14)
Mean maximum diameter of dominant follicle (mm)	12 – 14 (15)	6-7 (12, 31)	28 (30)	18-20 (14)
Average number of dominant follicles per wave	1-2	1-4 (31)	1-2 (33)	1 (14)
Number of follicular waves	2-3 (15, 19)	3-4 (12)	2 (major and minor) (13)	2- 3 (major and minor)
Seasonality	Non-seasonal	Seasonal (short-day) (34)	Seasonal (long-day) (13)	Non-seasonal
Litter size/multiple gestation	Monotocous	Mono or polytocous	monotocous	Mono or polytocous

## 3 Polyendocrine metabolic ovarian syndrome

PMOS is a complex syndrome that, in addition to reproductive impairment, is associated with several comorbidities, including obesity (20-80% of cases), hyperandrogenism (40-80%), metabolic dysfunction (50-80%), and increased cardiovascular disease risk (3). Infertility is 15 times more prevalent in women with PMOS than in age-matched controls, and the syndrome increases the risk of obstetric complications, psychological disorders and certain malignancies (3). The etiology of PMOS remains unknown: genetic factors account for approximately 10% of cases, implicating developmental and lifestyle influences as additional contributors (35). Diagnosis of PMOS requires identification at least two of three Rotterdam criteria (36, 37). Three principal pathogenic mechanisms have been proposed, which are not mutually exclusive: hyperandrogenism, ovarian dysfunction, and gonadotrophin derangement (**Figure 1**). Each is discussed below, with attention to how it relates to the farm animal models evaluated in following sections.

**Figure 1:**
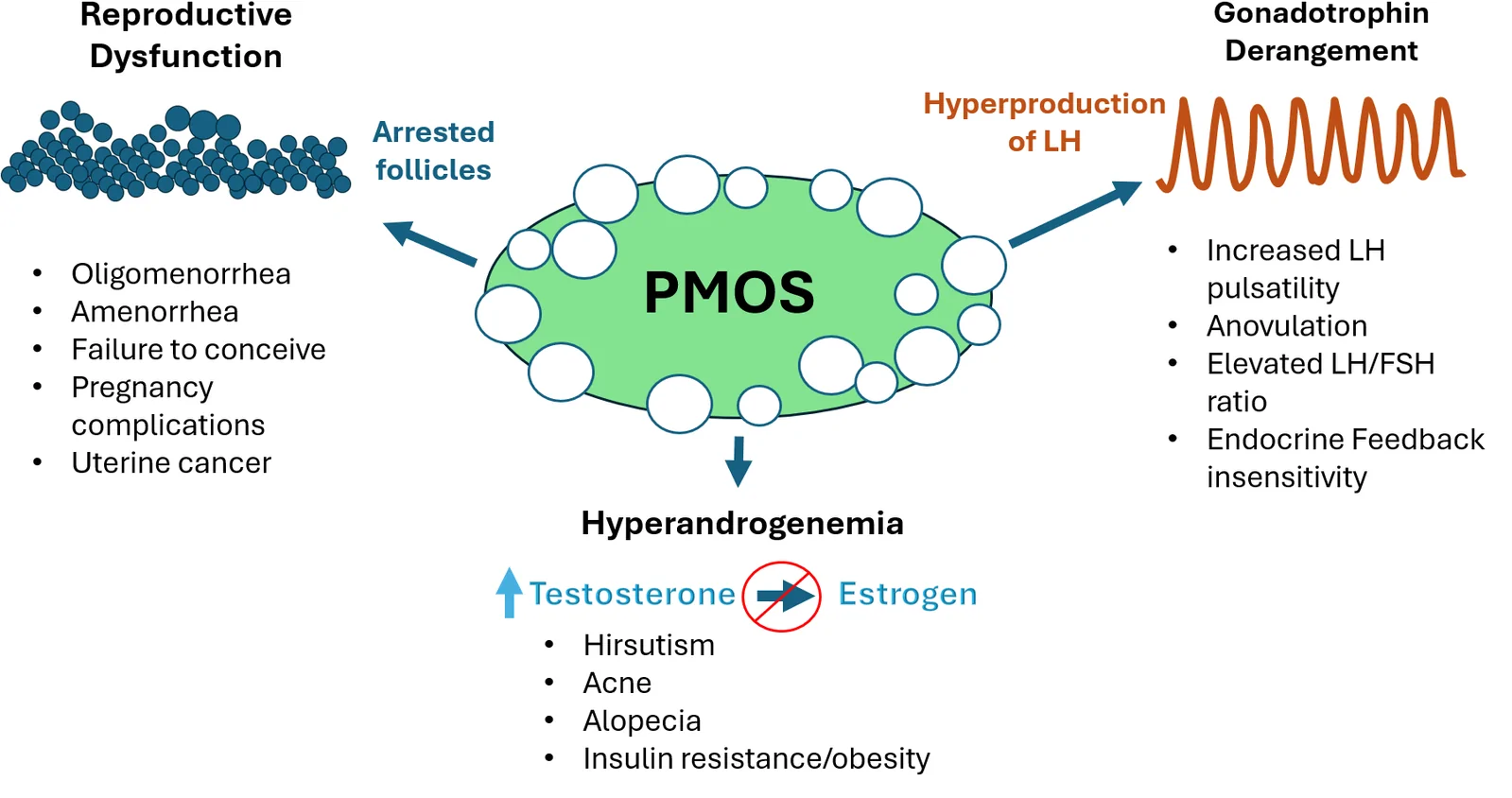
Schematic diagram representing the effects of polycystic ovarian syndrome. Reproductive dysfunction is characterized by disorganized ovarian folliculogenesis which leads to altered or absent menstrual cycles. Hyperandrogenemia results from a natural hyperproduction of androgens from theca cells in the ovary, and it leads to cosmetic and metabolic alterations. Gonadotrophin derangement occurs due to defective negative feedback at the hypothalamus with consequent endocrine dysregulation. These three pathways are likely integrated; however, the cause-effect relationships remain unknown.

### 3.1 Hyperandrogenism

Hyperandrogenism is the most diagnostically central feature of PMOS, though its phenotypic presentation varies substantially among patients. Ovarian-derived hyperandrogenism predominates in approximately 67% of cases, adrenal hyperactivity accounts for approximately 20%, and the remaining 13% cases display either both phenotypes or no detectable hyperandrogenism (38). Under normal conditions, ovarian steroids synthesis is governed by the two-cell two-gonadotrophin model (39). Cells in the theca interna express CYP11A1 and CYP17A1 and produce testosterone and androstenedione from circulating cholesterol under LH stimulation (40), while adjacent granulosa cells aromatize these androgens to estradiol and estrone via FSH-induced aromatase (CYP19A1) expression. The excess androgen production in PMOS likely reflects an intrinsic hypersecretory activity of theca cells that surpasses the aromatization capacity of granulosa cells. This notion is supported by studies of cultured theca interna cells from PMOS ovaries, which sustain hyperandrogenic activity compared to controls (41), whereas granulosa cell steroidogenesis does not differ between PMOS and control patients (42). Transcriptomic analyses of PMOS theca cells confirm significantly higher expression of LH receptor, StAR, CYP11A, and CYP17 compared to controls (43, 44). Aromatase expression is also reduced in PMOS theca cells, and follicular hyperandrogenism further suppresses aromatase mRNA and protein expression, creating a self-reinforcing androgenic intrafollicular environment (45). The intrinsic origin of thecal cell hyperactivity in PMOS patients remains to be elucidated.

### 3.2 Ovarian dysfunction

Amenorrhea, irregular menstrual cycles and anovulation in PMOS patients are direct consequences of ovarian follicular dysfunction. Within the follicular wave framework described in an earlier section, the arrested follicle development in PMOS corresponds to a “preselection” state (<10 mm of diameter), follicles that have been recruited but fail to progress through normal selection dynamics. This arrest is not absolute as ultrasonographic data indicate that 37-50% of amenorrheic women diagnosed with PMOS display follicular growth, selection of a dominant follicle and ovulation at least in some cycles (46, 47), suggesting impaired rather than absent selection capacity. When follicular selection does occur in POCS, wave dynamics are not altered in terms of growth rate or phase duration compared to control groups; rather, there is an irregular pattern of recruitment with variable timing of ovulatory dominant follicle emergence and occasional double ovulations (46). PMOS granulosa cells display lower atretic markers and longer survival capacity in culture than control patients (48, 49), indicating that the arrested cohort retains growth capacity but becomes refractory to the signals that normally guide follicular atresia. This notion is partially supported by studies in which providing exogenous support by administration of FSH promoted follicular growth past the 10 mm threshold reaching ovulatory sizes in 50% PMOS women (50), “surpassing” the follicular arrest phase.

Proposed intraovarian mediators of aberrant folliculogenesis in PMOS include follistatin and anti-Müllerian hormone (AMH). Follistatin, a glycoprotein produced in many tissues including ovarian follicles, suppresses FSH secretion by antagonizing members of the transforming growth factor beta (TGF-β) superfamily (51), is elevated in the circulation of PMOS patients compared to controls (52). These observations are consistent with paracrine inhibitory signaling contributing to follicular stagnation. AMH, a glycoprotein produced by granulosa cells of small growing follicles, normally suppresses primordial follicle activation and modulates FSH sensitivity in early antral follicles (53). AMH is consistently elevated in the blood of females diagnosed with PMOS (54) and has been proposed to contribute to impaired follicular selection, potentially in part through a neural pathway modulating GnRH neuron excitability (55), though the mechanistic basis of this pathway remains to be established. Farm animal models with documented alterations in intrafollicular AMH and follicular arrest, particularly hyperandrogenic sheep and cattle (56, 57) may provide tractable *in vivo* platforms for dissecting these intraovarian regulatory relationships.

### 3.3 Gonadotrophin derangement

Sustained elevation of LH pulse frequency and amplitude is a well-established hallmark of PMOS, documented across multiple phases of the menstrual cycle (58, 59). The LH hypersecretion likely reflects impaired negative feedback of ovarian steroids on hypothalamic GnRH output (60). The observations from a mouse study, where chronic chemogenetic activation of GnRH neurons produced a PMOS-like state, supports the idea that enhanced GnRH secretion plays a role in PMOS (61), while the primary upstream candidates are hypothalamic kisspeptin neurons of the arcuate KNDy network (62, 63). However, recent calcium-imaging studies in transgenic mice have questioned the centrality of KNDy dysregulation in rodent PMOS models (10), underscoring the need for complementary experimental models. As discussed below, prenatally androgenized sheep provide the most compelling large-animal evidence of gonadotrophin derangement, while preliminary data in letrozole-treated cattle promises a future bovine model.

## 4 Farm animal models of PMOS

Farm animal species offer two technical advantages over rodent models for studying the ovarian and endocrine features of PMOS. First, ovarian size is comparable to that of humans, enabling transrectal ultrasonography for non-invasive longitudinal assessment of follicular dynamics at a resolution not achievable in small animals (64). Second, body size permits repeated blood sampling over extended periods without compromising physiological variables, enabling characterization of pulsatile hormone secretion profiles across complete reproductive cycles (64). Together, these features make farm animals suited for studying the ovarian, endocrine and metabolic features of PMOS as an integrated phenotype. The following sections evaluate evidence from sheep, cattle, pigs and horse, organized around the three PMOS features identified in earlier section.

### 4.1 Sheep

The sheep has been more extensively used as a PMOS model than any other farm animal species. A foundational series of studies in the 1970s demonstrated that prenatal testosterone administration permanently disrupts adult reproductive cyclicity in sheep (65, 66), establishing the prenatal androgenization paradigm that has since generated the most comprehensive large-animal PMOS dataset (67). Two sheep models, the induced prenatal androgenization model and a naturally occurring hyperandrogenic breed, are discussed below.

Prenatal androgenization of Poll Dorset ewes leads to reproductive quiescence during the second breeding season and ablation of preovulatory LH surge response to a subcutaneous implant of estrogen in ovariectomized ewes, indicating a central defect in estradiol positive feedback on LH secretion (68). At the ovarian level, prenatal testosterone increased ovarian weight and volume at gestational day 140 compared to controls (69). Longitudinal ultrasonographic assessment over two breeding seasons revealed a lower proportion of ewes displaying normal estrous cycles, a marked increase in large follicles (>8 mm), and a reduction in medium (4–8 mm) and small antral follicles (3–4 mm) in androgen-treated animals compared to controls (70). Follicle lifespan was increased across all size classes, particularly in the 4–8 mm cohort, consistent with impaired follicular atresia (70). These alterations likely originate during early folliculogenesis: prenatal testosterone decreased primordial follicles at 10 months of age, but not in prenatal life at 90 or 140 days of gestation (69). Furthermore, prenatal treatment with testosterone increased growing follicles by at gestation day 140 and postnatally at 10 months of age (69), and produced a transient increase in small antral follicles between 18 and 26 weeks of age (71). These findings are consistent with increased primary follicle numbers in PMOS ovaries (72).

Luteal phase dysregulation is a consistent but complex finding in prenatally androgenized sheep. In one study, a prolonged luteal phase of up to 20 days was reported compared to 12 days in controls (70), while other studies have documented shortened estrous cycles (73). This apparent contradiction likely reflects dose- and timing-dependent effects of prenatal testosterone on steroid feedback mechanisms, which variably alter luteolysis timing and magnitude. Importantly, luteal phase abnormalities have not been documented in PMOS women (74), and this divergence should be acknowledged as a species-specific limitation of the prenatal androgenization model rather than a parallel to human PMOS.

At the neuroendocrine level, prenatally androgenized ewes exhibit altered organization of hypothalamic kisspeptin and KNDy neuronal networks, leading to impaired LH surge generation and disrupted GnRH pulsatility (75, 76). Prenatal testosterone alters the density and distribution of kisspeptin-immunoreactive neurons in the arcuate nucleus and anteroventral periventricular region, reducing the hypothalamic capacity to generate appropriate estradiol-positive feedback responses (67). Elevated LH pulse frequency has been documented in prenatally androgenized ewes under conditions of reduced progesterone feedback, paralleling the gonadotrophin hypersecretion observed in PMOS women (58, 59). These neuroendocrine features make the prenatal androgenization sheep model one of the most translationally relevant large-animal PMOS systems currently available, as it recapitulates ovarian, gonadotrophin, and hypothalamic features simultaneously.

A naturally occurring complement to the induced prenatal model is provided by a flock of Awassi sheep in Jordan with documented ovarian hyperproduction of androgens (56). This spontaneous phenotype allows examination of intraovarian androgen excess without exogenous hormonal manipulation. Awassi ewes with high androstenedione concentrations display 13.7-fold elevated follicular fluid androstenedione compared to controls, with only modest differences in estradiol. This intrafollicular androgen excess is associated with a paradoxical increase in primordial follicles and a substantial reduction in primary, secondary and antral follicle numbers, alongside dramatically increased expression of steroidogenic enzymes and LH receptor in theca cells (56). While the Awassi sheep does not fully recapitulate polycystic morphology, it provides a valuable model for studying the intraovarian consequences of chronic androgen excess on follicular development. Notably, prenatal androgenization and the Awassi model produce opposite effects on primordial follicle numbers — an increase and a decrease, respectively — a divergence that likely reflects differences in the timing, dose, and source of androgen exposure, and that merits dedicated mechanistic investigation (56, 77).

### 4.2 Cow

The bovine species combines well-characterized follicular wave dynamics, feasibility of longitudinal monitoring, and the existence of naturally occurring hyperandrogenic phenotypes, making it a valuable platform for studying PMOS-relevant ovarian mechanisms. Polycystic ovarian morphology resembling the human ultrasonographic phenotype is extremely rare in cattle; a single case report has documented bilateral polycystic ovaries in a Nellore cow (78), confirming this morphology does not arise naturally in this species. Research interest has consequently focused on naturally occurring hyperandrogenic phenotypes, follicular cystic disease, and variation in antral follicle count.

Naturally occurring hyperandrogenic cattle have been identified in beef and dairy herds in the United States and the Middle east (57, 79). These animals display repeatable, cycle-independent hyperproduction of androgens within antral follicles, with intrafollicular androstenedione and testosterone elevated 24.5- and 30.8-fold, respectively, in hyperandrogenic compared to controls (79). Theca cells from hyperandrogenic cows express significantly higher levels of STAR, LHCGR, CYP17A, CD68 and the macrophage marker CD68 compared to controls (57). Histological assessment reveals increased number of primary follicles but reduced secondary and antral follicular numbers in hyperandrogenic cows compared to controls (57, 80). Furthermore, *in vitro* cultured ovarian cortex from high androstenedione cows had intrinsic steroidogenic hyperactivity compared to controls (57). One important caveat of this model is that dominant follicle selection appears to proceed normally, and dominant follicle size does not differ from controls (79), indicating that the follicular arrest characteristic of human PMOS is not fully recapitulated.

A particularly translatable feature of the hyperandrogenic bovine model is the presence of ovarian stromal fibrosis. In PMOS women, ovarian interstitial fibrosis driven by increased TGF-β1 is a documented histological feature (81). Hyperandrogenic cows display both increased perifollicular collagen deposition and elevated CD68 expression, a macrophage maker sustained proinflammatory activity, in the ovarian cortex (57, 80). These parallel observations propose the hyperandrogenic bovine model as a uniquely informative system for investigating how ovarian stromal inflammation and fibrosis may contribute to follicular dysfunction in PMOS, a causal relationship that has not yet been established in human studies. A recently characterized bovine genetic variant, the Trio allele, produces co-dominant follicles and multiple ovulations per wave. Cows carrying this allele display altered follicular wave dynamics with simultaneous development of multiple dominant follicles, elevated circulating gonadotrophins, and a distinct hormonal profile compared to non-carriers (82, 83). While Trio cows do not model PMOS directly, they provide a naturally occurring large-animal system for investigating the mechanisms governing follicle selection and dominance. The very processes disrupted in PMOS may complement the understanding of multiple ovulation events and gonadotrophin interactions relevant to women.

Follicular cystic disease in cattle is characterized by the persistence of a large follicle (>25mm) in the absence of ovulation, or corpus luteum and prolonged dominance period, frequently associated with anestrus and infertility. Cystic follicles display altered intrafollicular steroid profiles, elevated progesterone and testosterone with reduced estradiol, compared to pre-ovulatory dominant follicles (84), and transcriptomic analysis has identified significant overlap in gene regulatory networks between bovine cystic follicles and polycystic ovaries from PMOS patients (85). Bovine follicular persistence models also provide a unique platform to evaluate the effect of stressors in anovulation, an insidious aspect of human diseases, including PMOS. Stressors such as hypoglycemia and inflammation are common in bovine anovulatory conditions, preventing gonadotrophin secretion, follicular function and ovulation (86, 87). While distinct differences exist between dairy cows and PMOS patients (i.e., high metabolic requirements vs obesity), these factors may be operating in a similar manner. Follicular cystic disease does not replicate the polycystic morphological pattern of PMOS, in which multiple small follicles are arrested rather than a single large structure persisting, but the anovulatory status and shared transcriptomic signatures suggest mechanistic parallels in follicular arrest pathways that merit further investigation.

Variation in antral follicle count (AFC) among individual cattle during the estrous cycle is a well-documented and highly repeatable phenotype (repeatability 0.95) that classifies cows into high- and low-AFC phenotypes not entirely dependent on circulating gonadotrophins (88). Importantly, high-AFC cows display approximately two-folds elevated circulating androgens compared to low AFC cows, and their theca cells demonstrate hyperresponsiveness to LH stimulation in androstenedione production (89). This AFC–androgen relationship in cattle may provide a mechanistic parallel for understanding why polycystic ovarian morphology, which reflects an elevated recruitable follicle pool, is associated with hyperandrogenism in PMOS patients. Whether AFC functions as a cause, consequence, or bidirectional modulator of ovarian androgen production in both species remains an important open question.

### 4.3 Sow

The pig is a valuable model for studying the intersection of metabolic disease and reproductive function, given well-established physiological and metabolic similarities with humans in cardiovascular function and glucose metabolism (90). The Ossabaw pig, a feral breed characterized by a thrifty-phenotype mutation conferring enhanced fat deposition, develops obesity and metabolic syndrome when fed a hypercaloric, high-cholesterol and high fructose diet, resulting in hyperinsulinemia, hyperglycemia, hypercholesterolemia and hypertriglyceridemia (91–93). The onset of diet-induced obesity in this model is accompanied by a 27% increase in estrous cycle length (92). Further characterization revealed elevated circulating androstenedione, dehydroepiandrosterone and progesterone in obese compared to lean Ossabaw pigs, alongside prevalence of cystic follicles and altered follicular populations during the luteal phase (94). Theca cells from obese Ossabaw pigs display increased steroidogenic activity under basal conditions and following LH and insulin stimulation, directly linking metabolic dysregulation to ovarian endocrine function (94).

Two limitations of this model are relevant to its use as a PMOS model. First, the polytocous nature of porcine reproduction, in which multiple follicles are simultaneously recruited and mature in parallel, makes direct comparison of follicular arrest dynamics with the monovulatory PMOS phenotype methodologically complex. Second, the obese Ossabaw pig does not appear display elevated LH pulse frequency and amplitude that is a defining gonadotrophin feature of PMOS in women (58, 59). Despite these limitations, the Ossabaw model offers a uniquely controlled system for investigating the causal relationships between metabolic dysfunction, altered ovarian steroidogenesis, and reproductive cycle disruption — mechanisms particularly relevant to the metabolic PMOS phenotype in obese women with insulin resistance. Whether induced models such as prenatal androgenization or aromatase inhibition produce a PMOS-like phenotype in pigs has not, to our knowledge, been systematically tested and represents an unexplored avenue.

### 4.4 Mare

Mare are the farm animal species with the closest resemblance to human ovarian function (29, 95, 96). In both mares and women, follicular development is organized into major and minor waves, with the dominant follicle from a major wave proceeding to ovulation and the timing of dominant follicle emergence during the mid-cycle major wave closely paralleling that of women (29). Despite this biological similarity, PMOS-like conditions are rare in mares (97), and the species remains the most underutilized farm animal in PMOS research relative to its translational potential.

The most relevant naturally occurring condition in mares is the hemorrhagic anovulatory follicle (HAF), in which a large preovulatory follicle hemorrhages internally without rupturing and the follicular wall subsequently luteinizes, producing a state of anovulation with extended lifespan and increased follicular diameter (98). The HAF display elevated androgen concentrations compared to preovulatory follicle in normal mares (99), paralleling the androgenic intrafollicular environment of PMOS. However, a notable discordance with the PMOS phenotype is the finding of reduced, rather than elevated, AMH concentrations in mares with HAF (100). Moreover, a human condition denominated luteinized unruptured follicles that parallels HAF has been described in women with notable morphological differences with PMOS (101). Since polycystic ovaries and elevated AMH are a consistent diagnostic correlate in PMOS women (54), this divergence warrants careful interpretation and should be considered a model limitation until mechanistically explained.

## 5 Conclusions and remaining questions

As summarized in **Table 2**, no single farm animal species fully recapitulates the complete PMOS phenotype as defined by the Rotterdam criteria. However, considered together, current evidence from sheep, cattle, pigs, and horses demonstrates that farm animals can model specific and mechanistically important components of PMOS in a manner that complements both rodent models and clinical research. The central strength of farm animal models is their capacity to interrogate ovarian and endocrine mechanisms *in vivo*, with the spatial and temporal resolution that longitudinal ultrasonographic monitoring and repeated blood sampling provide.

**Table 2:** Comparative summary of farm animal PMOS models.

Model	Type	Hyperandrogenism	Ovarian dysfunction	Gonadotrophin derangement	Metabolic features	Key limitations
Sheep — prenatal androgenization (65–70, 73, 75–77)	Induced	Intrafollicular; altered theca expression	Altered follicular populations; impaired selection; follicle lifespan increased	Impaired LH surge; elevated LH pulse frequency; altered KNDy networks	Insulin resistance documented	Luteal phase abnormalities not seen in PMOS women; dose/timing-dependent outcomes vary across studies
Sheep — Awassi (56)	Spontaneous	Intrafollicular androstenedione elevated; theca hyperactivity	Reduced antral follicles; increased primordial follicles (opposite to prenatal model)	ND	ND	No systemic hyperandrogenemia; gonadotrophin profile not characterized; primordial follicle changes are opposite to PMOS
Cow — hyperandrogenic (57, 79, 80)	Spontaneous	Intrafollicular androgens elevated; ovarian stromal fibrosis	Reduced antral follicle numbers; dominant follicle selection proceeds normally	ND	ND	No systemic hyperandrogenemia; follicular arrest not recapitulated; gonadotrophin profile not characterized
Cow — cystic follicular disease (84, 85)	Spontaneous/induced	Elevated intrafollicular testosterone (early cysts)	Single large anovulatory follicle; transcriptomic overlap with PMOS ovaries	Impaired LH surge generation	**~** Metabolic stress common precipitant	Does not replicate polycystic morphology (single large cyst vs. multiple small follicles); mechanistic overlap with PMOS incompletely defined
Cow — high AFC phenotype (88, 89)	Spontaneous	Elevated circulating androgens; theca LH hyperresponsiveness	High antral follicle count analogous to polycystic morphology	ND	ND	Not a disease state; mechanism of AFC variation unknown; gonadotrophin profile not characterized
Sow — Ossabaw obese (90–94)	Diet-induced	Elevated circulating androstenedione and DHEA; theca hyperactivity	Cystic follicles; disrupted follicular populations; extended cycle length	Not recapitulated	Obesity, insulin resistance, dyslipidemia (strong model)	Polytocous species complicates comparison with monovulatory PMOS; LH derangement not demonstrated; no wave-based follicular arrest
Mare — hemorrhagic anovulatory follicle (HAF) (98, 99)	Spontaneous	Elevated intrafollicular androgens in HAF vs. normal follicles	Single large anovulatory structure; extended follicle lifespan	ND	ND	Reduced AMH (opposite to PMOS women); rare; significantly understudied; gonadotrophin profile not characterized

AFC, antral follicle count; AMH, anti-mullerian hormone; DHEA, dehydroepiandrosterone; HAF, hemorrhagic anovulatory follicle; ND, not determined.

Hyperandrogenism, the most consistently observed PMOS feature across farm animal models, manifests primarily as an intrafollicular phenomenon in cattle and sheep rather than as systemic hyperandrogenemia as in PMOS women. This is a fundamental translational limitation: while ovarian theca cells are the source of excess androgens in both PMOS and farm animal models, the concentration of that excess within the follicular microenvironment rather than the systemic circulation means that downstream endocrine consequences may differ. Whether chronic intrafollicular androgen excess in naturally hyperandrogenic cattle or sheep produces measurable systemic hyperandrogenemia under specific physiological conditions remains to be determined, and resolving this question is important for establishing the degree of endocrine equivalence between species. The antral follicle count–androgen relationship in cattle represents a promising avenue: the two-fold elevation in circulating androgens in high-AFC cows, combined with theca cell LH hyperresponsiveness, more closely resembles the systemic endocrine signature of PMOS and warrants further investigation as a functional model.

Ovarian dysfunction is most comprehensively modeled by the prenatally androgenized sheep, which reproduces impaired follicular dynamics, altered follicular populations at multiple developmental stages, and defective LH surge generation in a manner that parallels polycystic ovaries. The naturally hyperandrogenic bovine model adds the unique dimension of ovarian stromal fibrosis, a feature of PMOS ovaries whose role in follicular arrest remains mechanistically undefined. Using the bovine model to determine whether and how ovarian stromal fibrosis impairs follicular development would address an important unanswered question in PMOS ovarian biology.

Gonadotrophin derangement is inadequately modeled by most current farm animal systems, representing the most critical gap in the large-animal PMOS literature. The prenatally androgenized sheep model provides the most relevant neuroendocrine data, including altered KNDy network organization and elevated LH pulse frequency. The letrozole model, which effectively recapitulates LH hypersecretion in mice (4) and has shown preliminary evidence of altered ovarian function in cattle (102), should be formally tested and validated in large animals as a priority. If confirmed, a letrozole-based large-animal model would provide the first farm animal system combining gonadotrophin derangement with experimentally tractable ovarian dynamics.

Three specific research directions emerge from this synthesis as highest priority. First, characterization of LH pulsatility in naturally hyperandrogenic cattle and sheep should be pursued as a short-term goal, as it would directly address whether these models recapitulate the neuroendocrine alongside the ovarian features of PMOS. Second, formal testing of aromatase inhibitor-based induction of PMOS in cattle and mares would determine whether the letrozole approach, successful in rodents, extends to monovulatory species with human-comparable ovarian dynamics. Third, the AFC–androgen relationship in cattle should be examined experimentally, including whether pharmacological reduction of AFC normalizes androgen production, to assess its utility as a functional endocrine PMOS model. In this regard, one approach to treat PMOS has been surgical ovarian wedge resection, reinforcing the idea that the size of AFC contributes to the development of PMOS. The use of farm animals combined with novel research tools such as gene editing will provide a powerful opportunity for inquiring the mechanistic pathways in health and disease. Together, these approaches would substantially advance the mechanistic understanding of follicular arrest in PMOS and improve the predictive value of large-animal models for developing novel diagnostics and therapeutic strategies.
